# Educational attainment and trajectories of cognitive decline during four decades—The Glostrup 1914 cohort

**DOI:** 10.1371/journal.pone.0255449

**Published:** 2021-08-02

**Authors:** Kristine Harrsen, Kaare Christensen, Rikke Lund, Erik Lykke Mortensen

**Affiliations:** 1 Department of Public Health, University of Copenhagen, Copenhagen, Denmark; 2 Danish Aging Research Center, Institute of Public Health, University of Southern Denmark, Odense, Denmark; 3 Department of Clinical Genetics and Department of Clinical Biochemistry and Pharmacology, Odense University Hospital, Odense, Denmark; 4 Center for Healthy Aging, University of Copenhagen, Copenhagen, Denmark; The University of New South Wales, Neuroscience Research Australia, AUSTRALIA

## Abstract

**Background:**

The potential association between level of education and age-related cognitive decline remains an open question, partly because of a lack of studies including large subsamples with low education and follow-up intervals covering a substantial part of the adult lifespan.

**Objectives:**

To examine cognitive decline assessed by a comprehensive clinical test of intelligence over a 35-year period of follow-up from ages 50 to 85 and to analyze the effect of education on trajectories of cognitive decline, including the effects of selective attrition.

**Methods:**

A longitudinal cohort study with a 35-year follow-up of community dwelling members of the Glostrup 1914 cohort. The study sample comprised 697 men and women at the 50-year baseline assessment and additional participants recruited at later follow-ups. Verbal, Performance, and Full Scale IQs were assessed using the Wechsler Adult Intelligence Scale at ages 50, 60, 70, 80, and 85. To be able to track cognitive changes between successive WAIS assessments, all IQs were based on the Danish 50-year norms. Information on school education was self-reported. The association between education and cognitive decline over time was examined in growth curve models. Selective attrition was investigated in subsamples of participants who dropped out at early or later follow-ups.

**Results:**

The trajectories for Verbal, Performance, and Full Scale IQ showed higher initial cognitive performance, but also revealed steeper decline among participants with a formal school exam compared to participants without a formal exam. Verbal IQ showed the largest difference in level between the two educational groups, whereas the interaction between education and age was stronger for Performance IQ than for Verbal IQ. In spite of the difference in trajectories, higher mean IQ was observed among participants with a formal school exam compared to those without across all ages, including the 85-year follow-up.

Further analyses revealed that early dropout was associated with steeper decline, but that this effect was unrelated to education.

**Conclusion:**

Comprehensive cognitive assessment over a 35-year period suggests that higher education is associated with steeper decline in IQ, but also higher mean IQ at all follow-ups. These findings are unlikely to reflect regression towards the mean, other characteristics of the employed test battery or associations between educational level and study dropout.

## Introduction

The increasing life expectancy and increasing elderly population [1,2] will lead to an increase in individuals with symptoms of age-related cognitive decline. The age at which normal age-related cognitive decline begins remains open for debate [3–5], but longitudinal studies suggest that substantial decline is rarely observed before the age of 60 [3,6], while some signs of decline, e.g. in processing speed and memory, may be observed in the 20s and 30s [4,7,8].

The consequences of cognitive decline vary and will depend not only on the timing of cognitive decline, but also on the cognitive domains that are most affected as well as the initial cognitive level from which the individual declines. Prevention of cognitive decline is obviously important, but few predictors of individual differences in trajectories and amount of decline have been consistently identified [9,10]. One of the most investigated and discussed potential predictors is educational attainment and length of education [11]. This is partly because educational attainment is known to correlate substantially with different measures of cognitive ability [12,13], but also because education has been linked to cognitive reserve theory [11]. Comprehensive measures of intelligence or cognitive ability tend to show substantial correlations with educational attainment over much of the lifespan [14,15], and low education is associated with increased risk of dementia [16]. However, the evidence on whether education moderates trajectories of cognitive decline is not consistent [11]. A recent meta-analysis found that associations between education and age-related cognitive decline are weak, non-significant and inconsistent with directions of associations differing among cognitive domains [17]. In meta-regressions the only significant predictor was length of the follow-up interval, but the direction of the associations differed among cognitive domains. However, many studies on cognitive decline have relatively short follow-up intervals [11], and it is possible that subtle effects of education on cognitive decline can only be reliably detected with follow-ups over larger parts of the lifespan than usually analyzed. Furthermore, a previous review [11] has also pointed out that effects of education on cognitive decline are under-researched in samples with low education [< 8 years]. Thus, there may still be a need to evaluate effects of education in studies including substantial low-education samples, even though Seblova et al. [17] did not find mean educational level to be a significant predictor in meta-regressions.

For global or composite measures of cognitive function there are studies reporting no association [18,19] and studies finding higher education to be associated with slower [20] or steeper decline [21,22]. Differences between domains of cognitive ability with respect to both timing and amount of cognitive decline are well-established and recent studies have tended to include cognitive tests presumably assessing specific cognitive functions from different cognitive domains. Thus, a study based on Whitehall II data assessed a range of cognitive functions to demonstrate decline at ages 45–49 [5], but used no global measure, and a recent study [23] analyzed the rise and fall of different cognitive abilities across the lifespan. However, in spite of the different trajectories of cognitive decline, there is now evidence that age-related changes in different cognitive domains are correlated and that the shared variance increases with age [24]. This suggests that comprehensive measures of cognitive abilities may be sensitive measures of effects of education on cognitive decline since education appears to affect a broad range of cognitive abilities, including crystallized and fluid abilities [14] and correlations with educational attainment tend to be high for broad, composite measures of cognitive ability.

The aim of the present study was to analyze associations between trajectories of cognitive decline in a sample characterized by a majority with low education and an unusual long follow-up interval. Thus, we examined cognitive decline assessed by a comprehensive clinical test of intelligence over a 35-year period of follow-up from ages 50 to 85 and analyzed the association between school education and trajectories of cognitive decline. The primary outcome was global measures of intelligence, but analyses of the included subtest scores were also conducted to further evaluate whether decline is global or limited to specific cognitive functions.

Since attrition from mortality or other factors is an important factor in longitudinal studies with long follow-up intervals [25,26], we also investigated the influence of selective attrition on the observed cognitive decline and on the relationship between education and cognitive decline.

## Materials and methods

### Study population

The study is part of the longitudinal studies of the Glostrup 1914 cohort, which was initiated to identify risk factors for coronary heart disease [27,28], but has also been much used to analyze cognitive aging [29–31]. At baseline 697 individuals born in 1914 and living in a suburban area west of Copenhagen completed the original version of Wechsler Adult Intelligence Scale (WAIS), one of the most widely used clinical tests of intelligence from which three global measures of intelligence can be derived [32]. The original study population was invited to all later follow-ups; in addition, new participants were recruited under the same criteria as the original cohort at the 70- and 75-year follow-ups, and in total 181 new participants contribute data to the IQ analyses. The analyses of WAIS IQs are based on all participants with information on WAIS IQs and education at the 50-, 60-, 70-, 80-, and 85-year assessments while the analyses of individual subtests also included data from the 75- and 90-year follow-ups.

### Cognitive ability

Wechsler Adult Intelligence Scale consists of 11 subtests: six verbal (Information, Comprehension, Similarities, Arithmetic, Digit Span, and Vocabulary) and five performance (Digit Symbol, Picture Completion, Block Design, Picture Arrangement, and Object Assembly) tests [32]. From the verbal and performance subtests, Verbal, Performance, and Full Scale standard WAIS IQs are constructed to obtain a mean of 100 and a SD of 15 for all age groups, and consequently they cannot be used to analyze age-related changes in intelligence. To be able to track IQ changes across assessments, the Danish 50-year norms were used to derive Verbal, Performance, and Full Scale IQs both at the 50-year examination and at all later follow-ups [33]. At the 50-, 60-, and 70-year follow-ups all participants in the IQ analyses completed all 11 subtests, but at the 80-year and in particular the 85-year follow-up some participants had missing data on one or more subtests. In these cases, IQs were prorated from the available subtest scores. Due to missing subtest scores, Performance and Full Scale IQs could not be calculated for one participant at the 50- and 60-year assessments, for 16 participants at the 80-year follow-up, and 13 participants at the 85-year follow-up.

### Education

Participants were asked about their school education at the 50- and 80-year assessments. As expected for Danes born in 1914 the vast majority only had basic school education (7 years) while a minority completed a formal exam after 10 or 12 years in primary and secondary school. Consequently, the participants were divided into two categories, those with more than 7 years of education and formal exam and those with 7 years or less education and without a formal school exam. This measure of education was chosen because only about 20–25% of the sample had a formal school exam and a large part of the participants–in particular women–had no post-school training or education. The majority of those with post school education had vocational training, and only about 10% had education at college or university level. At the 50-year exam the difference in mean Full Scale IQ between those with and without school exams was about 16 IQ points, corresponding to more than a theoretical standard deviation of 15 and a point-biserial correlation of 0.45.

### Data analyses

At each of the five follow-ups, observed means and SDs were calculated for the Full Scale, Performance, and Verbal IQs. ANOVA was applied to investigate the variance in scores of cognitive performance between the two educational groups defined by having a formal school exam vs. no formal school exam. Linear regression was used to analyze mean differences in cognitive performance across the follow-ups by taking repeated measurements into account (including a cluster-statement in the regression model) for both the full sample and for the subsamples defined by educational level. Follow-up age was included as a categorical variable. R-squared was used to measure the effect size for both education and age at follow-up.

To examine the trajectories of cognitive decline over time for each of the three WAIS IQs, Stata’s mixed procedure was used to analyze a basic growth curve model, including linear effects of age as both a fixed and random effect and assuming both random intercept and slope. Since decline in WAIS IQs were nonlinear, age squared was included, and the models additionally included sex, education and the linear interaction between education and age (preliminary analyses showed that the interactions between sex and age, between sex and education or between sex, education and age were non-significant). These analyses were repeated for all individual subtests and additionally for the IQs for a subsample comprising only individuals who participated in the original 50-year assessment.

A sensitivity analysis was conducted to evaluate how the length of the follow-up interval affected the ability to statistically detect any influence of educational attainment on decline in WAIS IQs. Thus, the main mixed model analysis was replicated for the 50- to 60-year data only, for the 50- to 70-year data and the 50- to 80-year data.

According to current Danish law all health science projects must be reported to and approved by the local scientific ethics committee (§ 14 in the law). However, the law specifies several exceptions to this general rule, such as 14.2 which states that projects based on questionnaires and/or register-data should only be reported and approved if the project also involves human biological material. Since the analyses do not involve biological material and none of the study participants are alive by now, this study does not require approval by the Danish scientific ethical committee system.

## Results

Table 1 shows the characteristics of participants at baseline and each follow-up in the IQ analyses. The percentages of men were higher than women in the first waves while the percentage of women and the percentage with a formal school exam were higher at the 85-year follow-up. However, this selective attrition was relatively moderate for both sex and education with the percentage of men dropping from 56 to 40 percent and the percentage with a formal school exam increasing from 21 percent to 24 percent.

**Table 1:** Analysis sample for the WAIS IQs*.

Variable	50-year baseline	60-year follow-up	70-year follow-up	80-year follow-up	85-year follow-up
**Number of participants**	697	551	141	349	163
Original 50-year sample**	697 (100%)	529 (96%)	141(100%)	208 (60%)	115 (71%)
**Sex, men (n, %)**	391 (56%)	308 (56%)	73 (52%)	166 (48%)	65 (40%)
**Having a formal school exam (n, %)**	147 (21%)	126 (23%)	30 (21%)	72 (21%)	39 (24%)

* Participants with WAIS test results and information on education from the 50-year baseline and follow-ups at 60, 70, 80, and 85 years.

** Subsamples of the original 697 participants in the 50-year assessment.

The analyses of individual subtests additionally included 193 participants from the 70-year assessment who were only administered Digit Span, Digit Symbol, Picture Completion and Block design. These analyses also included 268 participants from the 75-year assessment (Digit Span and Digit Symbol) and 109 participants from the 90-year assessment (Information and Digit Symbol). See details in supporting S1 Table.

Table 2 shows the means and SDs of Verbal, Performance, and Full Scale WAIS IQs both for the full sample and subsamples stratified by education. All three IQs declined significantly over time (p = < .001) in both the full sample and the categories defined by education. For the full sample the R-squared measures suggest that age explains substantially more variance in the Performance compared to the Verbal IQ, and for the subsamples age explains substantially more variance among participants with a formal school exam compared to those without an exam. The differences in R-squared for the two educational categories reflect differences in age-related changes, but in spite of these differences, analyses stratified on age show significantly higher mean IQs in the category with a formal school exam across all ages, including the 85-year follow-up.

**Table 2:** Mean (SD) for Verbal-, Performance-, and Full Scale IQ stratified on education.

	50-year baseline	60-year follow-up	70-year follow-up	80-year follow-up	85-year follow-up	R^2^	P-value^a^
**Verbal IQ**	Mean (SD)	Mean (SD)	Mean (SD)	Mean (SD)	Mean (SD)		
**Full sample**	98.0 (14.4)	98.8 (14.8)	96.4 (13.8)	89.6 (14.9)	88.4 (15.6)	0.07	<0.001
**Formal exam**	110.8 (12.0)	111.8 (11.8)	104.1 (13.2)	102.0 (13.9)	96.6 (17.0)	0.14	<0.001
**No formal exam**	94.5 (13.0)	95.0 (13.4)	94.3 (13.2)	86.4 (13.5)	85.9 (14.3)	0.07	<0.001
**R square**	0.21	0.23	0.09	0.18	0.09		
**P value**^b^	< 0.001	<0.001	<0.001	<0.001	<0.001		
**Performance IQ**							
**Full sample**	99.0 (14.5)	97.4 (14.0)	93.6 (14.4)	81.7 (13.8)	80.1 (13.5)	0.22	<0.001
**Formal exam**	109.2 (12.1)	106.2 (11.9)	98.4 (17.5)	88.6 (13.3)	86.5 (13.3)	0.32	<0.001
**No formal exam**	96.3 (13.8)	94.8 (13.5)	92.3 (13.2)	80.0 (13.4)	78.2 (13.0)	0.21	<0.001
**R square**	0.13	0.12	0.03	0.07	0.07		
**P value**^b^	<0.001	<0.001	0.04	<0.001	<0.001		
**Full Scale IQ**							
**Full sample**	98.8 (14.6)	98.5 (14.6)	95.1 (14.2)	85.3 (14.7)	84.1 (14.5)	0.14	<0.001
**Formal exam**	111.6(12.0)	110.6 (11.8)	102.2 (15.7)	96.1 (13.6)	92.1 (14.2)	0.25	<0.001
**No formal exam**	95.4 (13.4)	94.9 (13.3)	93.2 (13.2)	82.4 (13.6)	81.6 (13.7)	0.15	<0.001
**R square**	0.20	0.21	0.07	0.14	0.09		
**P value**^b^	<0.001	<0.001	0.002	<0.001	<0.001		

^a^ R square and p-value for differences in mean IQ scores between follow-ups.

^b^ R square and p-values for differences between participants with and without a formal school exam at each follow-up.

Similar analyses were conducted on all 11 WAIS subtests. For the full sample age explained considerably more variance in the performance subtests (range 9.0%; 16.9%) than the verbal subtests (range 2.2%; 6.2%), but on all subtests age explained more variance in the subsample with formal school exam.

**Fig 1:**
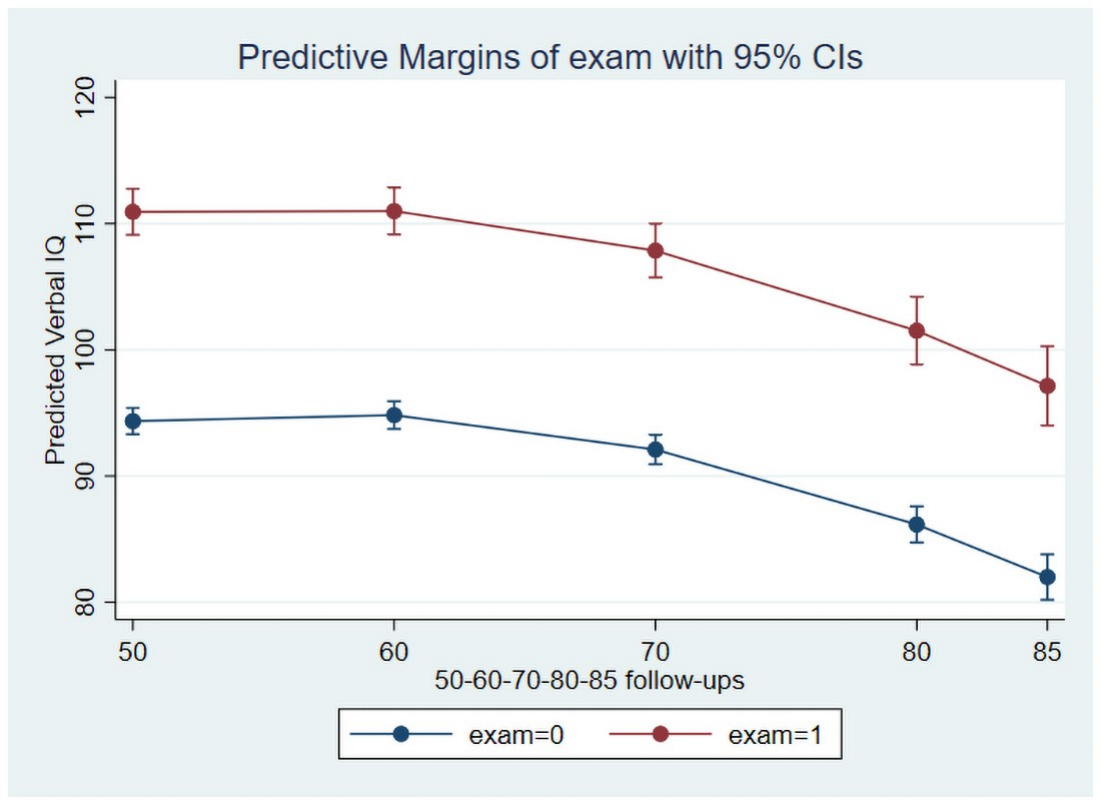
Decline of Verbal IQ stratified on education over a 35-year follow up.

**Fig 2:**
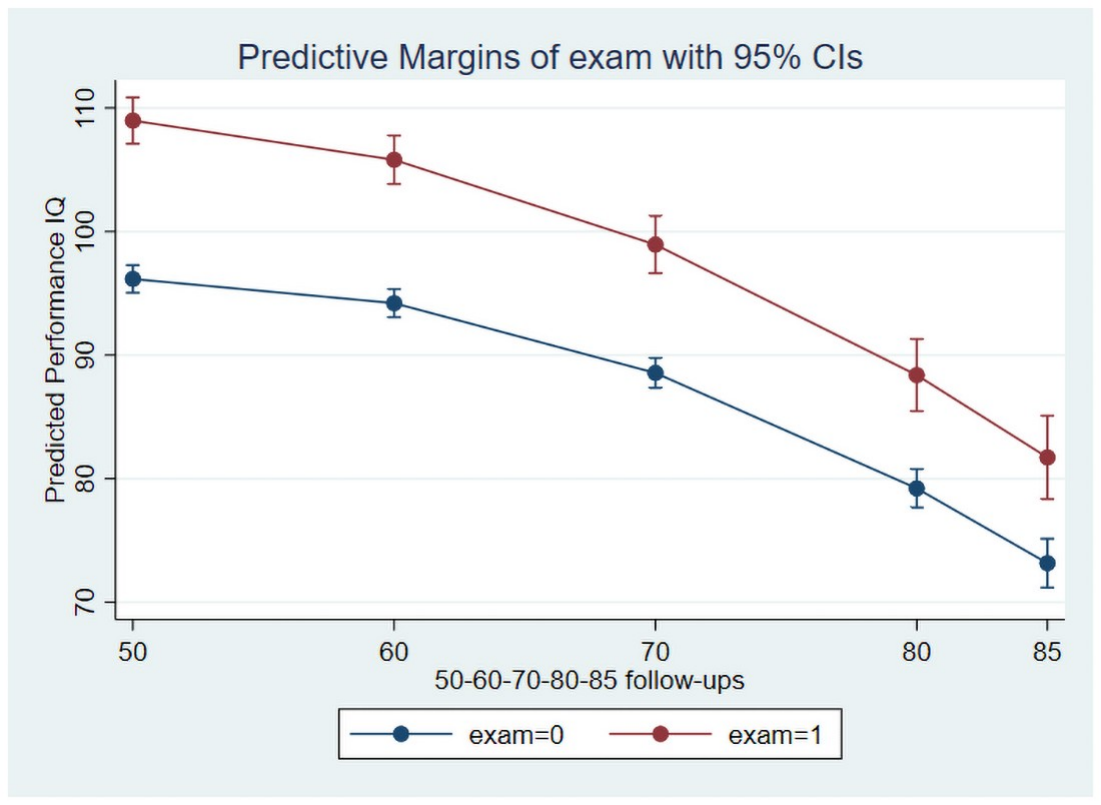
Decline of Performance IQ stratified on education over a 35-year follow up.

As preliminary analyses showed that the only significant interaction was between education and age at follow-up, the main growth curve model included age, age-squared, sex, education and the interaction between education and age. This model showed significant linear and quadratic effects of age, main effects of sex and education for all three IQs while the interaction between education and age was significant for the Performance and Full Scale IQs. Table 3 presents the results for this model, and Figs 1 and 2 illustrate the age trajectories for Verbal- and Performance IQs for participants with and without a formal school exam, respectively.

**Table 3:** Coefficients for growth curve models for Verbal-, Performance-, and Full Scale IQ*.

Variable	Verbal IQ Coefficient (95% CI)	Performance IQ coefficient (95% CI)	Full Scale IQ coefficients (95% CI)
**Age**	1.78 (1.46; 2.10)	1.78 (1.46; 2.10)	1.94 (1.66; 2.22)
**Age Squared**	-0.02 (-0.02; -0.01)	-0.02 (-0.02; -0.02)	-0.02 (-0.02; -0.02)
**Sex (female)**	-7.03 (-8.65; -5.41)	-5.96 (-7.65; -4.27)	-7.18 (-8.85; -5.52)
**Formal school Exam**	19.49 (14.52; 24.46)	19.08 (13.74; 24.42)	21.33 (16.50; 26.15)
**Formal school Exam x Age**	-0.06 (-0.14; 0.02)	-0.13 (-0.21; -0.04)	-0.10 (-0.18; -0.02)

*All main effects (Age, Age squared, Sex, and Exam) were significant at p < 0.001 while the significance levels for the interactions between Formal School Exam and Age were 0.18, 0.004 and 0.014 for the Verbal, Performance and the Full Scale IQs.

The predicted trajectories show higher initial cognitive performance, but also steeper decline among participants with a formal school exam compared with participants without an exam. The difference in decline between participants with and without a formal school exam is clearly stronger for the Performance IQ. Further analyses restricted to participants (n = 697) enrolled at baseline at age 50, thereby excluding the additional recruitment at ages 70 and 75, revealed no substantial difference in the results (Table 4).

**Table 4:** Original 50-year participants; coefficients for growth curve models for Verbal-, Performance-, and Full Scale IQ*.

Variable	Verbal IQ Coefficient (95% CI)	Performance IQ coefficient (95% CI)	Full Scale IQ coefficients (95% CI)
**Age**	1.81 (1.48; 2.14)	1.83 (1.50; 2.17)	2.01 (1.71; 2.31)
**Age Squared**	-0.02 (-0.02; -0.01)	-0.02 (-0.02; -0.02)	-0.02 (-0.02; -0.02)
**Sex (female)**	-7.10 (-8.89; -5.30)	-6.55 (-8-44; -4.67)	-7.49 (-9.33; -5.65)
**Formal school Exam**	18.63 (13.56; 23.69)	18.90 (13.40; 24.40)	20.88 (15.93; 25.84)
**Formal school Exam x Age**	-0.04 (-0.13; 0.04)	-0.12 (-0.22; -0.03)	-0.09 (-0.17; -0.01)

* All main effects (Age, Age squared, Sex, and Exam) were significant at p < 0.001 while the significance levels for the interactions between Formal School Exam and Age were 0.34, 0.011 and 0.032 for the Verbal, Performance and the Full Scale IQs.

**Fig 3:**
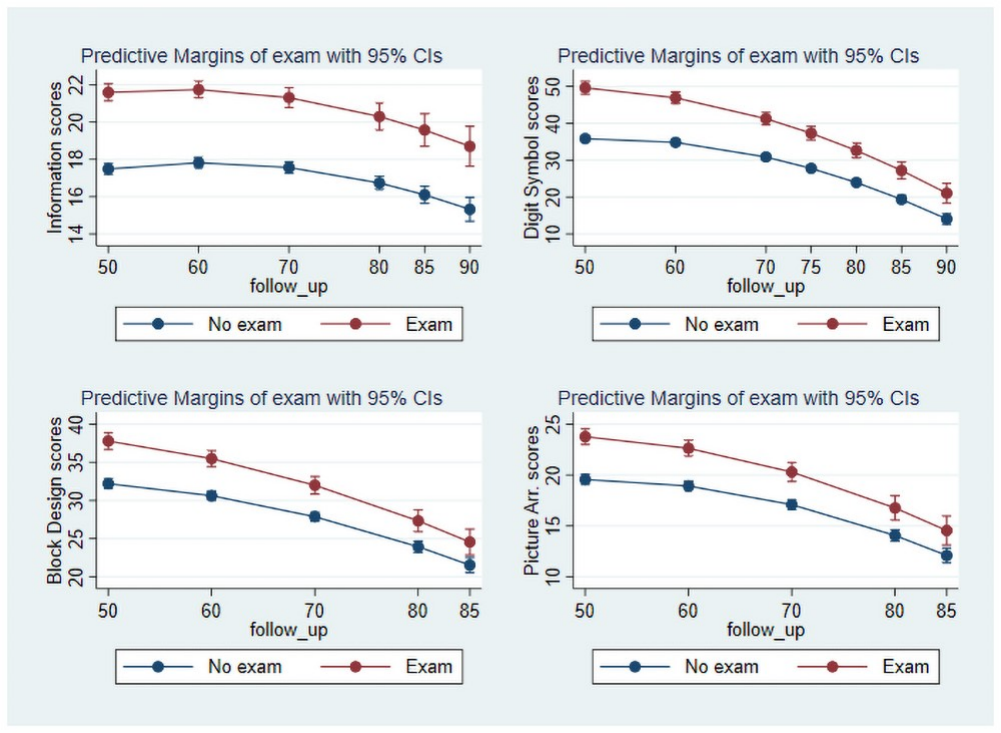
Decline on information, Digit Symbol, Block Design, and Picture Arrangement stratified on education over a 35- to 40-year follow-up.

In the growth curved models for individual subtests the interaction between education and age was only significant for Digit Symbol, Block Design, and Picture Arrangement (p< 0.001, p = 0.006, and p = 0.031). The estimated trajectories of performance on these subtests across the included follow-ups are presented in Fig 3 and compared with a typical verbal subtest such as Information. Results of the growth curve models are presented for all subtests in supporting S2 Table.

The sensitivity analyses showed no significant interaction between education and age when only the 60-year follow-up was included, but a significant interaction for the Performance IQ in the model including both the 60- and 70-year follow-ups. The Full Scale IQ was approaching significance in the model with 70-year follow-up, but was only significant when the 80-year follow-up was included. These results were similar for the full sample and the original sample only including participants in the 50-year study.

Table 5 presents mean Full Scale IQ for subsamples defined by the age at the last follow-up with administration of the WAIS irrespective of the reasons for dropping out (e.g. ill-health or being dead). For each age there is an IQ difference of about 6–7 points between the group who will drop out at the next follow-up and the group who participated in the 85-year assessment. Furthermore, for each age the difference between the participants who completed the next follow-up and those who did not was increasing (at age 50 this difference was 1.3 IQ points while it was 7.2 at age 80). For the 80-year old participants without the following 85-year follow-up, it is remarkable that the decline from the 50-year baseline is about 17–18 IQ points, corresponding to about 1.2 standard deviations on a scale with a theoretical IQ standard deviation of 15. For all subsamples the table shows minimal decline at age 60 compared to age 50.

**Table 5:** Full Scale IQ means for subsample completing the 50-, 60-, 70-, 80- and 85-year follow-ups, respectively.

Age at last follow-up	N	50-year baseline	60-year follow_up	70-year follow-up	80-year follow-up	85-year Follow-up	R squared ^a^	P value ^a^
**85 participation**	150	102.8	102.0	99.9	89.5	84.1	0.25	<0.001
**80 participation**	193	100.2	99.7	97.4	82.3		0.27	<0.001
**70 participation**	78	99.7	98.5	92.1			0.04	<0.001
**60 participation**	173	97.0	95.3				<0.01	0.003
**50 participation**	150	95.7						
**R squared** ^b^		0.03	0.03	0.06	0.06			
**P values** ^b^		<0.001	0.003	0.01	<0.001			

^a^ R squared and p values for differences in mean IQ scores between follow-ups.

^b^ R squared and p values for differences between groups at each follow-up.

The means in Table 2 suggests little decline in Full Scale IQ between ages 80 and 85, but the means in Table 5 suggest that this is because the 80-year subsample without the 85-year test strongly influenced the 80-year mean since the subsample participating in both assessments declined about 5 IQ points from 89.5 to 84.1. However, it is most likely that at every assessment those who will drop out of the study bring down the mean as a possible consequence of being less healthy and less cognitively intact than those remaining in the study.

**Fig 4:**
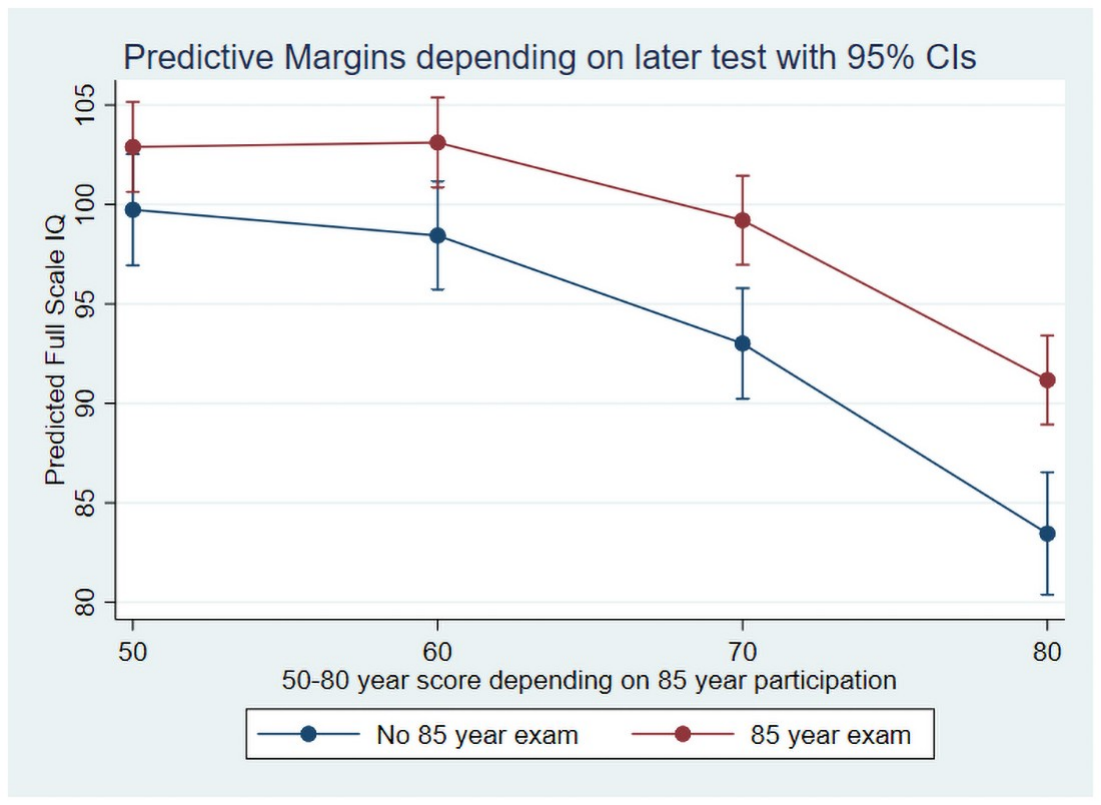
Full-scale IQ trajectories according to participation or non-participation in the 85-year follow-up.

Fig 4 shows Full Scale IQ trajectories for those who participated in both the 80- and 85-year assessment and those who only participated in the 80-year assessment. A growth curve model showed a significant interaction between participation group and age (p < 0.001) indicating that early dropout was associated with steeper decline.

A possible explanation for the interaction between education and age may be a relatively larger dropout of participants with steeper decline among those without a formal school exam. Since there was little decline at age 60 and the sample at age 70 was small, we focused on the 80- and 85-year assessments. The proportion of participants who did not complete the 85-year assessment was 54 and 61 percent among those with and without a formal school exam, respectively. The small difference was not statistically significant (p = 0.29).

## Discussion

Comprehensive assessment of the Verbal, Performance, and Full Scale IQs based on multiple administrations of the full WAIS showed that all three types of IQs significantly declined over time, both in the full sample and in subsamples defined by education. The analyses showed that age explained a larger part of the variance in Performance IQ compared to Verbal IQ and a substantially larger proportion of the variance in the subsample with a formal school exam compared to the subsample without an exam. When stratifying the analyses on age at follow-up the mean IQ among participants with a formal school exam was substantially higher than among participants without an exam up to and including age 85. In accordance with the observed means, a growth curve model showed a significant interaction between education and follow-up age for the Performance and Full Scale IQ and a non-significant trend for the Verbal IQ. These findings were corroborated by the analyses of individual subtests which showed significant interactions for Digit Symbol, Block Design and Picture Arrangement (the three subtests with most substantial age-related decline). The predicted trajectories revealed steeper decline among participants with a formal school exam, but also significantly higher cognitive performance from the initial 50-year assessment to the end of follow-up at age 85. The difference in IQ was largest between the two educational groups for Verbal IQ whereas for Performance IQ the data revealed a stronger interaction between education and age. Our results also confirmed the limited decline at age 60 and the previously reported substantial sex differences in performance on the Danish version of the WAIS [33,34].

Previous findings in this area have been inconsistent and most studies do not report significant differences in the rate of cognitive decline due to educational attainment [11,17]. Although two previous studies found steeper cognitive decline in Verbal Memory, Processing Speed, and Verbal Fluency among those with higher education [20,21], results were inconsistent in a recent population study finding higher education to be associated with less steep decline on three attention and memory tests, but steeper decline for three other tests within the same domains [35]. These results are corroborated by the inconsistent findings reported in a review [11] and in a recent meta-analysis [17] finding substantial heterogeneity and no significant association with education for decline in six cognitive domains, including global cognition. The inconsistent findings may be a consequence of methodological constraints such as shorter follow-up intervals and inadequate numbers of participants with low educational attainment in old age [11]. In this perspective, the present study adds to the literature on a possible impact of educational attainment on the trajectories of cognitive decline primarily because of the long follow-up interval covering the later part of the life-span and because the great majority of the participants only had seven years of school education. The sensitivity analyses showed the importance of the length of the follow-up interval and suggested that for a 50-year baseline the influence of educational attainment on trajectories of cognitive decline can only be detected with a 20-year or longer follow-up interval. Many studies of cognitive aging are based on follow-up intervals of less than 10 years, but at higher ages (> 70 years) decline may be steeper and more substantial with intervals of this length. A recent Danish study had an average follow-up interval of 41 years, but the average baseline age for this study was only 20 years [36]. Nevertheless, the study was able to demonstrate an association of longer education with steeper cognitive decline, but also observed an association between higher baseline scores and larger decline and found the influence of education to be reversed when adjusting for baseline intelligence scores [36]. The latter findings suggest that the main problem may not be to explain the association between higher education and steeper decline, but rather to explain the association between higher baseline cognitive scores and steeper decline. However, other studies have found inconsistent or weak associations between baseline cognitive scores and age-related decline [24,37].

The findings are somewhat ambiguous concerning the issue of whether school education is associated with decline in specific or global cognitive functions. Follow-up age seemed to be a stronger predictor of test performance on all subtests in the subsample with formal school exam, but significant interactions between education and age were only observed for three performance subtests. However, given the relatively small sample sizes at later follow-ups and the associated low statistical power, the findings may indicate an association between school education and global age-related intellectual decline that only became statistically significant for the most age-sensitive subtests. Global age-related intellectual decline would be in line with recent analyses suggesting that decline is associated across cognitive domains [24,37].

The differences in trajectories of cognitive decline between educational groups may reflect true differences in cognitive aging. Thus, the differences in the rate of decline were observed over long follow-up intervals and are only evident at relatively high age. This could indicate that educational differences in cognitive decline are associated with age-related physiological changes in the brain, which may impede higher levels of cognitive function more than the more basic levels associated with less school education. Whether this explanation is sufficient in a sample where higher educational level is likely to be associated with a healthier lifestyle is an open question.

This study and previous studies finding education to be associated with steeper decline apparently contradict most cognitive reserve theories assuming a protective influence of education on the effects of brain aging [14]. However, from a cognitive reserve perspective steeper decline in those with high education has been explained by assuming differences in timing of decline in those with low and high education. Thus, it has been assumed that those with low education have been affected by cognitive decline before enrollment in studies while those with high education will show more decline after enrollment because the effect of high education is to delay onset of cognitive decline. As previously pointed out [20], this is certainly a possibility in studies enrolling participants at high age at the first assessment. In our study we were unable to investigate the possibility of education related differences in decline in the 40s, but given the very moderate decline from the 50-year to the 60-year assessment, substantial differences in decline before the first assessment seems unlikely. In addition, education related differences in early decline can hardly explain the larger decline from baseline to follow-up associated with more years of education in a sample with baseline assessment at age 20 [36].

Repeated follow-ups may be associated with increasing practice effects, which are likely to have a relatively strong effect on the performance of individuals with higher educational attainment, since they might learn more from taking cognitive tests [38]. This may induce bias suggesting a protective effect of education in relation to cognitive decline. However, retest effects seem to be larger among younger individuals compared to older individuals [39] where the effect is estimated to be only 1/3 of the effect observed in young people [40]. Furthermore, in that study practice effects could no longer be observed after seven years in adults [40], and given the five to ten year intervals in the 1914 cohort it is likely that practice effects have relatively small impact on the results. This is corroborated by the observed substantial decline on the Performance IQ and subtests since practice effects are usually larger on this part of the WAIS [31,33].

The observed steeper decline may also be an artefact related to the quality of the assessment battery. First, higher educational level was associated with higher initial performance, and regression towards the mean might explain steeper decline in the subsample with a formal school exam. However, this seems unlikely because of the high reliability of the WAIS (Wechsler reported a reliability of 0.97 for the Full Scale IQ [32]) and because the interaction between school education and age at follow-up was observed over several follow-ups and was strongest for the Performance IQ with the smallest baseline difference in IQ level between educational groups. Second, time is a factor in the scoring of the Digit Symbol, Block Design and Picture Arrangement subtests, but it remains an open question whether age-related slowing of processing speed plays a role in the observed interaction between school education and age. A previous study of the 1914 cohort suggested that processing speed only played a limited role in the decline observed from the 50-year to the 70-year assessment [33]. Third, the fact that the curves tend to meet at the later follow-ups might be a test artefact reflecting a floor effect for the group without a formal school exam. This is, however, unlikely for a comprehensive cognitive battery such as the WAIS with eleven subtests and composite IQ scores. Fourth, there is the possibility that a relatively larger proportion of participants with poor health and steep decline dropout of the study in the subsample without a formal school exam. Because of the larger dropout, the remaining participants in this subsample would be expected to show less decline than observed in the subsample with a formal school exam. However, there were no substantial differences in attrition between those with and without formal school exams, and thus selective attrition is not a likely explanation of the differences in trajectories of cognitive decline.

Selective attrition is a recognized problem in most longitudinal studies and is often not well accounted for. Individuals with higher cognitive abilities more often enroll and remain in longitudinal studies [41,42]. In this study, participation at higher follow-up ages was systematically related to both higher initial cognitive performance and less steep age-related cognitive decline. Thus, a significant interaction showed that individuals who participated in the 80-year examination but dropped out before the final 85-year examination had steeper cognitive decline compared to those who completed all examinations.

### Strength and limitations

The major strengths of the present study are the length of the follow-up and multiple assessments, the comprehensive WAIS assessment of cognitive ability and cognitive change, and the relatively large sample with low education. The long, prospective follow-up makes it possible to observe cognitive changes over a 35–40 year period and to analyze potential interactions with educational level over this interval and at high ages. Cognitive decline is a progressive long-term process and consequently findings in studies on cognitive decline with shorter follow-up intervals [11] should be interpreted cautiously, in particular studies of cognitive domains showing marginal decline over 5–10 years [43]. A previous review has pointed to the lack of studies of cognitive decline in samples with low education [11]. The great majority of our initial study sample comprised individuals with low education and no formal school exam, and as a result a relatively large sample without a formal school exam participated in the later follow-ups.

There are some important limitations of the present study. One is a relatively small sample size, which became even smaller due to dropout. This may have reduced the statistical power, in particular with respect to the detection of all possible interactions. Another significant limitation may be the dichotomization of education with a cut-off under and above 7 years of formal school education. However, this dichotomization was chosen because the great majority of the cohort only had 7 years of school education and only vocational training if any post-school education at all. It is an unavoidable limitation that sensorimotor impairments become more frequent with increasing age and may have resulted in artificially degraded cognitive test scores for the oldest age groups. Finally, the study included no independent assessment of dementia, which might have influenced the observed decline, particularly at the later follow-ups. However, it is unlikely that participants with advanced dementia would be able to complete the comprehensive WAIS testing and more likely that dementia cases may be part of the explanation of the association between steeper decline and non-participation in later follow-ups. Other factors may be health problems or mortality leading to steeper decline. Evidence of terminal decline has previously been observed in the 1914 cohort [33].

Since the members of the cohort were all born in 1914 in Denmark, it is likely that they grew up under conditions that were specific for that time period and country, and consequently, it is an open question whether the results can be generalized to younger generations and other countries. Current school and post-school education are obviously not comparable to the beginning of the 20^th^ century. Higher education is much more prevalent in younger generations and there may be more variance in educational attainment. This raises the issue of whether relative educational differences within generations influence trajectories of cognitive decline or whether an absolute level of exposure to educational curricula is necessary to influence cognitive aging. Flynn effects are well-documented in Denmark for the last 60 years [44], and consequently cognitive decline will start from a higher cognitive level in younger generations. Whether this higher level influences trajectories of cognitive decline is an important issue, in particular since education is strongly associated with cognitive ability, and the present study observed steeper decline for the higher educated.

## Conclusion

While recent reviews [11] and meta-analyses [17] suggest weak or inconsistent associations between education and trajectories of cognitive decline, the present findings with comprehensive cognitive assessment over four decades suggest that higher education is associated with steeper cognitive decline, but in spite of the difference in trajectories, higher mean IQ was observed among participants with a formal school exam across all ages, including the 85-year follow-up.

We believe that these findings do not reflect regression towards the mean or other characteristics of the employed test battery. Furthermore, analyses of participation and study dropout indicate that the steeper decline in the high education subsample does not reflect selective attrition.

## Supporting information

S1 Table Analysis sample for the 1914 cohort. Participants with WAIS subtest results and information on education from the 50-year baseline and follow-ups at 60, 70, 75, 80, 85 and 90 years of age.
(DOCX)

S2 Table Growth curve models for age, sex and education predicting cognitive change in 11 WAIS subtests. * p < 0.05; ** p < 0.01; *** p < 0.001.
(DOCX)
