# Supplementary material for: Educational attainment and trajectories of cognitive decline during four decades—The Glostrup 1914 cohort
Source: PLoS One. 2021 Aug 2;16(8):e0255449. doi: 10.1371/journal.pone.0255449 (PMC8328320; doi:10.1371/journal.pone.0255449)
Supplement: S1 Table — Participants with WAIS subtest results and information on education from the 50-year baseline and follow-ups at 60, 70, 75, 80, 85 and 90 years of age. (DOCX) [file pone.0255449.s001.docx]

| Variable | 50-year baseline | 60-year follow-up | 70-year follow-up | 75-year^2^ follow-up | 80-year^2^  Follow-up | 85-year follow-up | 90-year follow-up |
| --- | --- | --- | --- | --- | --- | --- | --- |
| Number of participants | 698 | 551 | 334 | 268 | 349 | 163 | 109 |
| Original 50-year sample (n, %)^3^ | 698 (100) | 529 (96) | 334 (100) | 166 (62) | 189 (54) | 115 (71) | 63 (58) |
| Sex, men (n, %) | 391 (56) | 308 (56) | 181(54) | 129 (48) | 166 (48) | 65 (40) | 37 (34) |
| Formal exam (n, %) | 147 (21) | 126 (23) | 74 (22) | 49 (18) | 72 (21) | 39 (24) | 21 (19) |
