# Supplementary material for: Educational attainment and trajectories of cognitive decline during four decades—The Glostrup 1914 cohort
Source: PLoS One. 2021 Aug 2;16(8):e0255449. doi: 10.1371/journal.pone.0255449 (PMC8328320; doi:10.1371/journal.pone.0255449)
Supplement: S2 Table — * p < 0.05; ** p < 0.01; *** p < 0.001. (DOCX) [file pone.0255449.s002.docx]

| Variable | Age | Age^2^ | Sex (female) | Exam (Exam) | Exam x Age |
| --- | --- | --- | --- | --- | --- |
| Verbal subtests | **IQ coefficient**  **(95% CI)** | **IQ coefficient**  **(95% CI)** | **IQ coefficient**  **(95% CI)** | **IQ coefficient**  **(95% CI)** | **IQ coefficient**  **(95% CI)** |
| Information | 0.35*** (0.24 ; 0.47) | -0.003*** (-0.004 ; -0.002) | -2.78*** (-3.24 ; -2.32) | 5.01*** (3.38 ; 6.64) | -0.02 (-0.05 ; 0.01) |
| Comprehension | 0.11 (-0.00 ; 0.23) | 0.001** (-0.002 ; -0.001) | -1.36*** (-1.76 ; -0.95) | 2.51** (0.94 ; 4.07) | 0.01 (-.02 ; 0.03) |
| Arithmetic | 0.39*** (0.29 ; 0.48) | -0.003*** (-0.004 ; -0.003) | -1.73*** (-2.09 ; -1.37) | 23.68*** (2.37 ; 4.98) | -0.02 (-0.04 ; 0.00) |
| Similarities | 0.58*** (0.45 ; 0.72) | -0.005*** (-0.006 ; -0.004) | -1.32*** (-1.79 ; -0.84) | 3.81*** (1.97 ; 5.65) | -0.01 (-0.04 ; 0.02) |
| Digit Span | 0.10*** (0.04 ; 0.15) | -0.001*** (-0.001 ; -0.000) | -0.01 (-0.20 ; 0.18) | 0.95** (0.25 ; 1.66) | 0.01 (-0.00 ; 0.02) |
| Vocabulary | 1.08*** (0.81 ; 1.35) | -0.010*** (-0.012 ; -0.008) | -2.91*** (-4.17 ; -1.64) | 11.50*** (7.63 ; 15.4) | -0.02 (-0.08 ; 0.04) |
| Performance subtests | |  |  |  |  |
| Digit Symbol | 1.53*** (1.28 ; 1.77) | -0.015*** (-0.017 ; -0.013) | 0.69 (-0.62 ; 2.01) | 22.31*** (17.39 ; 27.24) | -0.17*** (-0.24 ; -0.10) |
| Picture Completion | -0.51*** (0.41 ; 0.62) | -0.004*** (-0.005 ; -0.004) | -2.42*** (-2.80 ; -2.03) | 3.45*** (2.09 ; 4.81) | -0.02 (-0.04 ; 0.00) |
| Block Design | 0.49*** (0.26 ; 0.73) | -0.006*** (-0.008 ; -0.004) | -3.07*** (-4.03 ; -2.11) | 9.25*** (5.92 ; 12.6) | -0.07** (-0.13 ; -0.02) |
| Picture Arrangement | 0.60*** (0.37 ; 0.82) | -0.006*** (-0.008 ; -0.004) | -2.32*** (-3.03 ; -1.61) | 6.72*** (3.95 ; 9.48) | -0.05* (-0.10 ; -0.01) |
| Object Assembly | 0.65*** (0.37 ; 0.93) | -0.007*** (-0.009 ; -0.005) | -1.78*** (-2.66 ; -0.89) | 3.09 (-0.56 ; 6.74) | -0.01 (-0.08 ; 0.05) |
